# The Test-Retest Variability of the COMPlog System in Participants with Induced Non-Normal Visual Acuity

**DOI:** 10.22599/bioj.127

**Published:** 2019-04-11

**Authors:** Mun Wei Kan, Anne Bjerre

**Affiliations:** 1 Royal Free London NHS Foundation Trust, GB; 2 University of Sheffield, GB

**Keywords:** computerised COMPlog, test-retest variability, Bangerter foils, computerised vision test

## Abstract

**Aim:**

The aim of this study was to determine and compare test-retest variability (TRV) of the computerised visual acuity (VA) COMPlog system on participants with normal vision and non-normal vision induced by bangerter foils (BFs).

**Methods:**

Twenty adult volunteers with VA of 0.100 logMAR or better in each eye and no eye conditions were included. Monocular VA data using the COMPlog system under five conditions—with plain Plano glasses (visually normal condition) and four pairs of Plano glasses with BF strengths of 0.6, 0.3, 0.2 and 0.1 (induced non-normal vision conditions)—were collected on two separate visits. To reduce bias, the eye tested and order of the BFs assessed were randomised. Data comparison was analysed using 2-factor ANOVA and paired t-tests and Bland Altman analysis to assess TRV.

**Results:**

Mean VA score from the two visits was –0.072 ± 0.1 logMAR for Plano, 0.106 ± 0.1 logMAR for BF 0.6, 0.428 ± 0.1 logMAR for BF 0.3, 0.662 ± 0.09 logMAR for BF 0.2 and 0.850 ± 0.08 logMAR for BF 0.1. As BF density increased, VA score significantly worsened (p < 0.0001). Overall mean VA score from the first and second visit was 0.410 ± 0.4 logMAR and 0.379 ± 0.4 logMAR, respectively. This improvement was significant (p < 0.009). The 95% limits of agreement of the VA scores between testing conditions had a range of ±0.120 to ±0.220 logMAR.

**Conclusions:**

Increase in BF strength led to a worsened VA score. However, the COMPlog TRV under the visually normal and induced non-normal vision conditions were within a similar range (±0.120 to ±0.220 logMAR). VA significantly improved on the second visit, suggesting a possible learning effect, which could have a clinical impact.

## Introduction

Detecting a true change in visual acuity (VA) is of great importance in ophthalmic care. This can be made difficult by test-retest variability (TRV), which can result in differences in VA scores when there is no genuine change. Computerised VA tests are becoming increasingly popular in clinic, and the benefits of using these in comparison to hard-copy test charts have been recognised (Laidlaw et al. 2007).

Previous studies (Laidlaw et al. 2007; Shah et al. 2012) have compared the TRV of the computerised COMPlog (using letters and Kay pictures) with the ETDRS in amblyopic children and both visually normal adults and those with ocular disease. Retesting of VA was carried out immediately after the initial testing. In the adult group, the TRV ranged from ±0.10 logMAR to ±0.14 logMAR and ±0.12 logMAR to ±0.16 logMAR using the computerised COMPlog and ETDRS, respectively. In the child participants, TRV ranged from ±0.12 logMAR to ±0.16 logMAR and ±0.12 logMAR to ±0.14 logMAR using the computerised COMPlog and ETDRS, respectively. TRV of all participant groups were similar, although a lower range was found in the child participants compared to the adults.

One study (Stewart et al. 2006) conducted retesting four to five weeks after the initial VA test, comparing the computerised ‘Staircased procedure’ to the ETDRS on visually normal children (mean age: 6.7 ± 1.1 years). No significant difference was found between the VA scores obtained at the two visits, and the TRV were similar (±0.15 logMAR for the Staircased procedure and ±0.13 logMAR for the ETDRS).

Bokinni et al. (2015) compared the computerised COMPlog with the ETDRS in participants diagnosed with age-related macular degeneration (AMD) and found a TRV of ±0.13 logMAR with both tests when presenting five-letters-per-line on the computerised COMPlog. The TRV (±0.17 logMAR) was slightly higher when the computerised COMPlog presented three-letters-per-line (Bokinni et al. 2015).

Noushad, Thomas & Amin (2012) investigated TRV using the ETDRS chart presenting five-letters-per-line and a modified logMAR chart presenting three-letters-per-line in participants with nonmacular disease and found TRV to be similar presenting five-letters-per-line (±0.10 logMAR) and three-letters-per-line (±0.08 logMAR).

Bangerter foils (BFs) have been used in several studies to induce VA deficits (McCulloch et al. 2011; Bowrey et al. 2015; Odell et al. 2008). Progressive degradation of VA was found with each subsequent filter density, although it has been suggested that the density of BF may not be consistent throughout the entire filter; therefore, it may be possible to search for a clearer portion of each BF to view through (Odell et al. 2008). The mean degradation in VA of non-amblyopic eyes using BF 0.3 and BF 0.2 was 4.8 logMAR and 5.11 logMAR respectively (Rutstein et al. 2010).

The studies described above report comparable TRV between VA tests, suggesting similar repeatability. However, TRV cannot be compared reliably as the ocular statuses of the participants were not consistent. Some VA retest assessments were conducted immediately after the initial test, and this is not comparable to a true clinical situation where retest would be on a separate occasion. Differing optotypes were also presented to the participants, which may have had an influence on TRV. Monocular VA has not been randomised in a number of previous studies (Laidlaw et al. 2007; Shah et al. 2012; Stewart et al. 2006), which may have resulted in bias. Due to these factors, it is difficult to apply the findings of previous research to a wider population.

In this research, the computerised COMPlog was chosen due to its increasing popularity in both clinical and research use. Visually normal participants were chosen to eliminate any differing statuses in ocular disease, which could pose as an extraneous variable. BFs were chosen as a means to induce standardised non-normal VA. VA test and retest assessment was conducted on two separate occasions, rather than during one visit. This was done to simulate a true clinical situation.

The purpose of this study was to investigate TRV of a computerised logMAR VA measurement system, termed COMPlog, on young adult participants with normal vision and non-normal vision induced by BFs. The VA scores obtained at the two visits were also compared.

## Methods

Ethical approval of this study was gained from the ethics committee in the Academic Unit of Ophthalmology and Orthoptics at the University of Sheffield.

### Participants and inclusion criteria

Participants were given a participant information sheet with full details of the study process to read before deciding whether to take part, and informed consent was gained from all of the participants. There were 20 adult participants (mean age 20 ± 1.2 years, range 19–23) recruited. Of those, 14 (70%) were female and 6 (30%) were male. The inclusion criteria of this study were the following: a VA level of 0.100 logMAR or better wearing contact lenses or unaided, no manifest strabismus, no latent strabismus measuring more than 10 prism diopters, a minimum stereopsis score of 150 seconds of arc, and no history of any eye conditions. There were 4 (20%) participants who wore contact lenses throughout the study, and 16 (80%) of the participants took part in this study unaided.

### Equipment

The computerised COMPlog consists of a PC, a 21-inch 1600 × 1200 resolution LCD flat panel secondary monitor and a software programme running within the Microsoft dotnet® framework. It consists of two phases: range finding and thresholding. During the range finding phase, approximate threshold is identified by presenting sequentially smaller single letters. Then the thresholding phase follows, starting at 0.2 logMAR larger than the range finding result. This phase consists of presentation of sequential lines of letters surrounded by a crowding box. Letters are not repeated on the same line. Line size increment in this phase is set at 0.1 logMAR. If a letter is identified incorrectly, then sequentially larger lines are presented until an entire line is correctly read. The programme descends to threshold, and it only presents lines of each size of letters once.

In this study, Sloan letters were presented with five letters on one line. They were spaced half a letter width apart and surrounded by a crowding box. The termination criterion was set at all fives letters on one line. The COMPlog was calibrated to a testing distance of 4M.

Other equipment used in this study included an occluder, a Frisby stereoacuity test (Stereotest Ltd, Sheffield, UK), a horizontal and vertical prism bar (Luneau SAS, Pont-de-l’Arche, France) and a stopwatch. Five pairs of Plano glasses were also used. Four different levels of BFs (Reyser Optik AG, St. Gallen, Switzerland) were selected with the nominal grades of 0.6, 0.3, 0.2 and 0.1 to induce a wide range of visual acuity. They were cut to size and adhered to four pairs of Plano glasses using water, and these were the induced non-normal vision conditions. One pair of Plano glasses was left clear, and this was the visually normal condition.

### Avoiding bias

In order to reduce bias, one eye of each participant was assessed throughout the study, with the same eye being assessed during the two visits. The left eye of 10 (50%) of the participants was assessed, whilst the right eye of the other 10 (50%) of the participants was assessed. The eye that was tested in each participant was decided randomly by picking out of a hat.

To prevent order effects, the order in which the different grades of BFs were used to induce non-normal vision in the participants was randomised. This was decided in advance using a counterbalanced measures design for four conditions (Shuttleworth 2009).

### Experimental procedure

This experiment was conducted in two phases. It was carried out in the same location under consistent lighting conditions and by the same examiner each time.

#### Phase 1 (first visit)

During phase one of this study, each participant was given a participant information sheet to read. The participant information sheet contained information about the study, including a detailed explanation of what each phase involved and the possible disadvantages and adverse effects in taking part. It was made clear to each participant that all of the information collected would be kept confidential and that they had the right to withdraw from the experiment at any time should they wish. Following this, the participant was given the chance to ask any questions. If the participant was willing to proceed, then they were asked to sign a consent form.

Participants were assessed to determine whether they met the inclusion criteria. If so, the study proceeded. The participant put on a pair of Plano glasses, and an occluder was used to occlude the lens of the eye that was not being tested. Each participant sat at a testing distance of 4M. They were asked to read the letters out loud, and the examiner input the responses into the computerised COMPlog as either correct or incorrect accordingly. When unsure of the letters, the participants were encouraged to guess. The monocular VA score obtained by each participant whilst wearing Plano glasses was recorded, and then the participant was given a 20-second break (timed by a stop watch), during which they were advised to close their eyes. After the 20-second break, a pair of Plano glasses with BF over both lenses was put on instead of the plain Plano glasses (the strength of BF used depended on the predetermined order of BF strength testing) and an occluder was used to occlude the lens of the eye that was not being tested. The monocular VA of the participant was tested again. The VA score was recorded, followed by another 20-second break.

This was repeated until all four different strengths of BF were used to induce non-normal visual acuity, indicating the end of phase one.

#### Phase 2 (second visit)

The participant was asked to return for phase two of the experiment at a similar time on a separate occasion within 12 days (mean 4). During phase two of the experiment, the same eye of the participant assessed during phase one of the experiment was assessed again. The same testing procedure from phase one commenced, but this time without the need to check whether the participant fulfilled the inclusion criteria.

### Statistical analysis

First, the data collected from this study was examined to determine whether it was normally distributed. The mean, standard deviation, standard error and range were calculated. A two-factor repeated measures ANOVA was conducted to determine whether the different strengths of BF and the visit during which VA was tested had an effect on the VA scores obtained. A paired-samples t-test was conducted to compare the VA scores obtained with Plano and each BF during the first and second visit and to compare the VA scores obtained during the two visits under the five testing conditions. A Pearson product-moment correlation coefficient was computed to assess the relationship between the VA scores obtained during the first visit and the second visit, under each testing condition. Finally, a Bland Altman analysis was conducted on the VA scores obtained during the first visit and the second visit under each testing condition. The 95% limits of agreement of each set of data were calculated using the following formula: 1.96 × Standard deviation (SD) of the difference.

## Results

The data collected was normally distributed. The mean, standard deviation (SD) and range values of each data set are displayed in Table 1.

**Table 1:** The mean, standard deviation (SD) and range of the VA scores (logMAR) obtained by the participant during the first and second visit with Plano, Bangerter foil (BF) 0.6, BF 0.3, BF 0.2 and BF 0.1.

	Bangerter Foil (BF) Strength									

	Plano		BF 0.6		BF 0.3		BF 0.2		BF 0.1	

Visit	First	Second	First	Second	First	Second	First	Second	First	Second

**Mean VA (logMAR)**	0.053	–0.091	0.137	0.074	0.447	0.408	0.651	0.673	0.868	0.831
**SD**	0.102	0.100	0.105	0.090	0.108	0.105	0.107	0.084	0.082	0.080
**Range**	0.380	0.380	0.380	0.340	0.380	0.340	0.480	0.340	0.280	0.300

**Figure 1:**
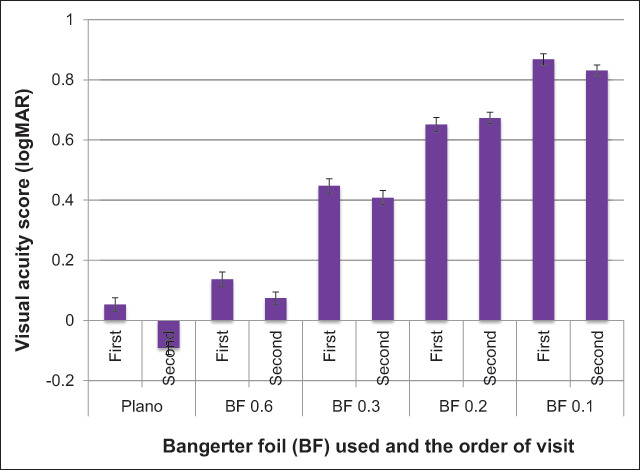
A bar graph showing the mean and standard error of the VA scores (logMAR) obtained by 20 participants with normal vision (Plano) and non-normal vision induced by different grades of Bangerter foils (BF) (BF 0.6, BF 0.3, BF 0.2 and BF 0.1) during two separate visits.

Figure 1 displays the mean VA score obtained under each testing condition.

The VA scores obtained varied between each testing condition. The VA scores obtained during the two visits increased from BF 0.6 to BF 0.1, as the BF density increased. The BF effect on the VA scores obtained during the first and second visit was significant, F (4, 19) = 661.182, p = <0.0001.

On paired t-test analysis, a significant difference was indicated in the VA scores obtained during the two visits between each BF, with a p value of <0.0001 for each.

There was a significant difference in the VA scores obtained with Plano first visit and Plano second visit (t (19) = 2.7, p = 0.0141), BF 0.6 first visit and BF 0.6 second visit (t (19) = 2.9, p = 0.0101) and BF 0.1 first visit and BF 0.1 second visit (t (19) = 2.6, p = 0.0181). These results suggest that when the participants had normal vision (Plano), the VA scores obtained during retesting were better than the first time they were tested. This finding was also the case when BF 0.6 and BF 0.1 were used to induce non-normal vision. There was no significant difference in VA score during the two visits when BF 0.3 was used to induce non-normal vision (t (19) = 1.737, p = 0.0986). Further, when BF 0.2 was used to induce non-normal vision, the VA scores obtained during the second visit appeared to worsen in comparison to the first visit; this difference was not significant (t (19) = –0.884, p = 0.3875). The interaction of BF with visit was significant (F (4, 19) = 2.77, p = 0.03).

A Pearson product-moment correlation coefficient was computed to assess the relationship between the VA scores obtained during the first and the second visit under each testing condition. A significant correlation between the VA scores obtained during the first and second visit with Plano, BF 0.6, BF 0.3 and BF 0.1 can be seen in Table 2. The correlations between VA scores obtained at the two visits with BF 0.2 were weaker, indicating a greater possibility that the results obtained were due to chance.

**Table 2:** A table displaying the correlation coefficient r-values and P-values of each testing condition during the two visits, significant at alpha = 0.05 level.

	r-value	P-value

**Plano**	0.81	<0.0001
**BF 0.6**	0.5	0.03
**BF 0.3**	0.56	0.01
**BF 0.2**	0.34	0.14
**BF 0.1**	0.69	0.009

A Bland Altman analysis was conducted on the VA scores obtained during the first and second visit with Plano, BF 0.6, BF 0.3, BF 0.2 and BF 0.1, and the 95% limits of agreement between each sets of data were calculated using the formula 1.96 × Standard deviation (SD) of the difference (Table 3). The 95% limits of agreement indicate the test-retest variability (TRV). The higher the TRV, the poorer the repeatability and reliability of the VA score.

**Table 3:** Bland Altman analysis of the visual acuity scores (logMAR) at visit one and visit two using the computerised COMPlog system, with Plano, Bangerter foil (BF) 0.6, BF 0.3, BF 0.2 and BF 0.1.

	Plano	BF 0.6	BF 0.3	BF 0.2	BF 0.1

**Bias (Mean)**	0.038	0.063	0.039	–0.022	0.037
**SD**	0.063	0.099	0.1	0.111	0.064
**95% Upper limits**	0.161	0.256	0.236	0.196	0.162
**95% Lower limits**	–0.085	–0.13	–0.158	–0.24	–0.088
**TRV (1.96 × SD)**	±0.123	±0.193	±0.197	±0.218	±0.125

## Discussion

The aim of this study was to determine and compare the TRV of the computerised COMPlog in young adult participants with normal vision and when BFs were used to induce non-normal vision in the same participants. The VA scores obtained during the two visits were also compared.

The mean VA scores measured with BF 0.3 (0.447 logMAR and 0.408 logMAR) was comparable to previous studies (Odell et al. 2008; Rutstein et al. 2010) that found mean VA scores of 0.440 logMAR and 0.500 logMAR, suggesting similar BF degradation. However, the VA scores measured with BF 0.1 (0.868 logMAR and 0.831 logMAR) were slightly better than the mean VA score (0.930 logMAR) reported by a previous study (Odell et al. 2008), suggesting slightly less BF degradation of VA. The mean VA scores found with BF 0.2 (0.651 logMAR and 0.675 logMAR) suggested more degradation of VA when compared to the mean VA (0.570 logMAR) found by a previous study (Odell et al. 2008). In general, increasing the density of the BF worsened the VA. This finding agrees with previous literature, which reports progressive degradation of VA with each subsequent BF (Odell et al. 2008).

Mean VA scores improved from visit one to visit two. This suggests that a learning effect may occur when using the computerised COMPlog. This is in agreement with a previous study (Stewart et al. 2006) that also found a significant improvement in VA on retesting. This is important to note in a clinical setting, where it is imperative to detect true changes in VA. It should also be taken into account that the participants may have searched for a clearer portion of each BF to view through on retesting under the induced non-normal conditions, which would have led to an improved VA score (Odell et al. 2008). The computerised COMPlog system randomises the letter optotypes presented during VA testing and thus reduces memory bias.

The TRV found in this study under Plano, BF 0.6, BF 0.3 and BF 0.1 testing conditions were within a similar range to the TRV found in previous studies. The TRV (±0.19) calculated with BF 0.3 (mean baseline VA 0.447 logMAR and 0.408 logMAR) was similar to the TRV (±0.14 and ±0.16) found by a previous study (Shah et al. 2012) when comparing participants with a similar baseline mean VA (0.440 logMAR and 0.480 logMAR). TRV of the computerised COMPlog under each testing condition were within 2 logMAR lines, except when BF 0.2 was used. This finding is in agreement with that reported by previous studies (Bokinni et al. 2015; Laidlaw et al. 2007; Noushad et al. 2012; Shah et al. 2012; Stewart et al. 2006). With BF 0.2, one participant achieved a better VA score during initial testing, with a 4 logMAR line decrease on retesting. This may be due to the participant viewing through a clearer portion of the BF during the initial VA test, a possibility suggested by Odell et al. (2008).

As different grades of BFs were used to induce non-normal vision, there was effectively a large range of baseline VA levels (range –0.072 logMAR to 0.850 logMAR) that TRV of the computerised COMPlog was calculated upon. This means that the TRV calculated may be applied to a wider range of patients in clinic who have differing levels of VA. The TRV of the computerised COMPlog was lowest with the normal vision condition (Plano), with a mean VA of 0.053 logMAR and –0.091 logMAR; this finding was comparable to a previous study (Laidlaw et al. 2007). This suggests that poorer levels of VA may result in greater variability.

However, BFs were used to induce non-normal vision on participants with normal vision during this study; therefore, it is difficult to make a direct comparison to previous research that investigates TRV on participants with reduced VA due to ocular disease. It was also difficult to ensure that degradation of VA using BFs was invariable from visit one to visit two, as it has been previously suggested that the density of BF may not be consistent throughout the entire filter (Odell et al. 2008). Although VA test and retest was carried out on separate occasions in this study, the interval between test and retest (mean four days) did not approximate the average time between VA tests in a clinical situation (six to eight weeks) and therefore reduces the applicability of the findings to a true clinical scenario. Although four (20%) participants wore contact lenses in order to correct refractive errors throughout this study, the refractive errors were not taken into account, and this may have had an effect on the findings. Another limitation of this study is the relatively small number of participants recruited. It may also have been useful to measure the TRV using the gold standard ETDRS using the same method in order to allow comparison.

Further research could be carried out to investigate the TRV of VA scores on the computerised COMPlog system in younger children participants with genuine ocular conditions, such as amblyopia, with retesting carried out at six to eight week intervals. This would produce results more applicable to a true clinical scenario. It would be interesting to research the TRV of children participants with different types of amblyopia to investigate whether this has an effect on TRV. The effect of refractive error on TRV could also be investigated.

## Conclusion

Good repeatability was found using the computerised COMPlog system to assess VA in visually normal and induced non-normal vision conditions in young adults. The TRV was within 2 logMAR lines, except when using BF 0.2, where the TRV was just over 2 lines. The computerised COMPlog system is a valid method of VA testing, displaying similar TRV to that found by other studies. A significant improvement was found in VA scores during retesting, which suggests that a learning effect may be present. Awareness of this finding is important when assessing patients clinically.
